# IEGS-BoT: An Integrated Detection-Tracking Framework for Cellular Dynamics Analysis in Medical Imaging

**DOI:** 10.3390/biomimetics10090564

**Published:** 2025-08-24

**Authors:** Shuqin Tu, Weidian Chen, Liang Mao, Quan Zhang, Fang Yuan, Jiaying Du

**Affiliations:** 1 College of Mathematics and Informatics, South China Agricultural University, Guangzhou 510642, China; 2 Undergraduate School of Artificial Intelligence, Shenzhen Polytechnic University, Shenzhen 518055, China; 3 College of Mechanical Engineering, Dalian University of Technology, Dalian 116024, China; 4 College of Computer Science and Electronic Engineering, Hunan University, Changsha 410082, China

**Keywords:** multiple object tracking (MOT), YOLO11n, IEGS-YOLO, cell tracking, BoT-SORT

## Abstract

Cell detection-tracking tasks are vital for biomedical image analysis with potential applications in clinical diagnosis and treatment. However, it poses challenges such as ambiguous boundaries and complex backgrounds in microscopic video sequences, leading to missed detection, false detection, and loss of tracking. Therefore, we propose an enhanced multiple object tracking algorithm IEGS-YOLO + BoT-SORT, named IEGS-BoT, to address these issues. Firstly, the IEGS-YOLO detector is developed for cell detection tasks. It uses the iEMA module, which effectively combines the global information to enhance the local information. Then, we replace the traditional convolutional network in the neck of the YOLO11n with GSConv to reduce the computational complexity while maintaining accuracy. Finally, the BoT-SORT tracker is selected to enhance the accuracy of bounding box positioning through camera motion compensation and Kalman filter. We conduct experiments on the CTMC dataset, and the results show that in the detection phase, the map50 (mean Average Precision) and map50–95 values are 73.2% and 32.6%, outperforming the YOLO11n detector by 1.1% and 0.6%, respectively. In the tracking phase, using the IEGS-BoT method, the multiple objects tracking accuracy (MOTA), higher order tracking accuracy (HOTA), and identification F1 (IDF1) reach 53.97%, 51.30%, and 67.52%, respectively. Compared with the base BoT-SORT, the proposed method achieves improvements of 1.19%, 0.23%, and 1.29% in MOTA, HOTA, and IDF1, respectively. ID switch (IDSW) decreases from 1170 to 894, which demonstrates significant mitigation of identity confusion. This approach effectively addresses the challenges posed by object loss and identity switching in cell tracking, providing a more reliable solution for medical image analysis.

## 1. Introduction

Cell detection and tracking task plays a crucial role in medical image analysis, enabling dynamic monitoring of cell behaviors such as proliferation, migration, and division [1]. These capabilities are vital for clinical applications like assessing cancer treatment efficacy [2,3]. Traditional cell image analysis is dependent on manual labeling and observation. This way is laborious, susceptible to errors, and has a negative impact on efficiency and accuracy [4,5].

In recent years, the development of deep learning has provided a new way to overcome the above bottleneck [6,7], and automated cell detection and tracking based on computer vision has become an essential component of biomedical research. In cell detection, Chen et al. [8] proposed a novel task decomposing and cell comparing network for cervical lesion cell detection, which achieved an AP@0.75 of 25.5%. Guo and Zhang [9] employed YOLOv5-ALT for blood cell detection, which integrated the SE attention mechanism into the backbone network and utilized an improved loss function EIOU. The method obtained a precision of 97.9%, a recall of 93.5%, and a mAP@0.5 of 97.4%. Ikeda et al. [10] used YOLOv5 for training and detection on cytology images prepared using two distinct liquid-based cytological processing techniques, achieving an F1 of 84.6%. Xie et al. [11] introduced an end-to-end dual-branch-based fully convolutional neural network (DB-FCN), which was evaluated on three datasets, achieving F1 of 89.7%, 89.7%, and 87.0%, respectively, demonstrating excellent generalization capabilities. Sazak and Kotan [12] utilized YOLO11 on the BCCD dataset for detecting red blood cells, white blood cells, and platelets, achieving a mAP@0.5 of 93.8%. Chang et al. [13] modified the original YOLOv5 architecture by replacing residual blocks with SENet modules to enhance crucial features and suppress low-level feature interference, resulting in a precision of 90.0%, recall of 88.0%, F1 of 89.0%, and mAP of 78.0%. Chen et al. [14] developed a multi-scale perceptual MSP-YOLO detection model incorporating a super-resolution reconstruction branch, which was designed to enable precise and efficient cell detection in complex vaginitis diagnosis scenarios. This method achieved F1 of 33.9% for clue cells and 54.3% for trichomonads. Tarimo et al. [15] proposed a hybrid architecture that combines the strengths of YOLO and vision transformer (ViT), effectively integrating convolutional operations with Transformer-based methods for enhanced object detection and classification performance in medical imaging analysis. Haq et al. [16] designed a deep learning-based algorithm for the efficient detection and counting of colorectal cancer cells (HT-29). Liao et al. [17] presented HistoMoCo method to generate models with enhanced image representations and initializations for OSCC detection in histopathological images. However, all the above studies relied on static images for cell detection and lacked the biomimetic mechanisms for sustained category tracking, a critical gap for dynamic analysis.

As an extension of the detection pipeline, cell tracking requires advanced processing of temporal and spatial dependencies in image sequences. This involves not only the identification of individual cells in single frames but also the establishment of identity correspondence across frames. Scherr et al. [18] developed a graph-based matching strategy that effectively handles cell tracking scenarios. Sixta et al. [19] designed a unified segmentation-detection-tracking framework implicitly defined through the stationary distribution of Markov chain Monte Carlo algorithms. Wang et al. [20] presented a deep reinforcement learning approach for inter-frame detection object association. The cost matrix is constructed by comprehensively integrating various target features to forecast the distribution of association solutions. Panteli et al. [21] introduced a simple end-to-end cascaded neural network architecture that can effectively simulate the motor behavior of biological cells and predict collision and division events. Chen et al. [22] proposed a unified end-to-end deep learning framework integrating cell segmentation and tracking, where cell detection and segmentation are performed through the current instance seg-mentation method and cell tracking is realized by integrating the Siamese network. Ben-Haim and Raviv [23] proposed an end-to-end deep learning framework for the detection of complete cell trajectories in high-throughput microscopy sequences. All the above studies have made some progress in cell tracking task; however, these methods still have limitations under complex cell scenes.

In real scenarios, intense motion of many cells and mutual occlusion often occur, and this situation is similar to the complex scenarios handled by multiple object tracking (MOT) technology. MOT is typically divided into two primary technical approaches: tracking-by-detection (TBD) and joint detection and tracking (JDT). The TBD algorithm is divided into two phases: detection and tracking [24]. In the first phase, a detector predicts the object of interest in each frame. In the second phase, a tracker uses the detection results as inputs to establish the trajectory of the object between frames by employing specific association strategies. The JDT method, on the other hand, fuses detection and tracking into a single framework [25], which reuses the features extracted from the detector, and the tracker can directly utilize the appearance features of the object output from the detector, avoiding the process of receiving the detection results from the tracker. Compared with JDT method, TBD has better tracking performance. Therefore, this TBD technique has been widely used in a variety of downstream tasks, including video surveillance, intelligent transportation, autonomous driving, military space, etc., demonstrating a strong capability to adapt to complex scenes.

In recent years, some researchers have begun to introduce TBD technology in the cell tracking field. For example, Anjum and Gurari [26] conducted experiments on the CTMC dataset using Tracktor and DeepSORT frameworks, demonstrating superior performance over traditional cell tracking algorithms. Toubal et al. [27] utilized EDNet and M2Track for cells, which demonstrated the potential of combining algorithms across domains.

Inspired by the above studies, this study introduces the TBD framework to the field of cell tracking. We propose an improved MOT method named IEGS-BoT to detect and track cell movement under complex backgrounds. First, to solve the problems of missed detection, false detection, and loss of tracking due to blurred boundaries, IEGS-BoT adopts an enhanced iEMA module. It is constructed by the efficient multi-scale attention (EMA) module [28] and the inverted residual mobile block (iRMB) [29]. Secondly, IEGS-BoT replaces the traditional convolution of the neck network with GSConv [30] to reduce the computational complexity. Finally, BoT-SORT [31] is selected as the tracker for the irregular division behavior of cell tracking tasks. This integrated biomimetic framework translates three core principles of biological vision—multi-scale perception, computational efficiency, and identity persistence—into an engineered solution for clinical cell analysis.

The main contributions of this study are as follows:

(1) We propose an enhanced MOT algorithm IEGS-YOLO + BoT-SORT, named IEGS-BoT, to detect and track cell movement for microscopic video sequences.
(2) To tackle the challenges of detecting small cells under complex backgrounds, we propose IEGS-YOLO detector, an enhanced cell detection network based on YOLO11n.
(3) The IEGS-YOLO integrates the iEMA module to increase model stability. Meanwhile, it uses a lightweight and efficient convolutional structure named GSConv module to reduce model parameters and computational complexity.
(4) We evaluate the performance of several mainstream trackers on a public dataset. BoT-SORT is ultimately selected and combined with the IEGS-YOLO detector for cell tracking and trajectories analysis.

## 2. Methods

**Figure 1:**
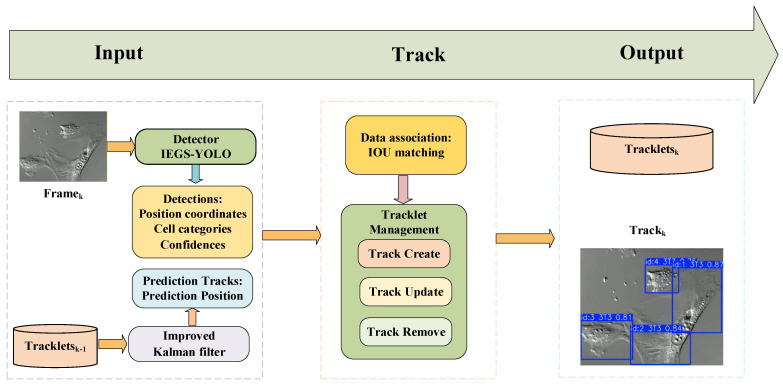
The structure of IEGS-BoT.

In this study, we propose a cell-tracking IEGS-BoT algorithm based on IEGS-YOLO detector and BoT-SORT tracker. The structure of IEGS-BoT is shown in Figure 1. Initially, IEGS-YOLO is used to detect each cell and obtain its detection box, category, and confidence in each video frame. The IEGS-YOLO reduces model parameters and enhances the model’s ability to recognize cell boundaries under complex backgrounds. Subsequently, the BoT-SORT tracker is employed to track each cell, which significantly improves the accuracy and robustness of cell tracking by integrating motion models, appearance information, and camera motion compensation techniques.

### 2.1. IEGS-YOLO Detector

We develop IEGS-YOLO based on YOLO11n, which is the smallest variant of the YOLO11 series for its fewer parameters, lower computational cost, and small model size. The YOLO11 architecture consists of four primary components: the input layer, Backbone, Neck, and Head. Initially, the input layer receives the raw image data and conducts preliminary processing. Its key role is to convert the data into a format compatible with the network, including resizing and normalizing the image for feature extraction and object detection through subsequent layers. Next, the Backbone network extracts the features such as edges, texture, and shape from the input image. Subsequently, the Neck network receives the feature maps obtained from the Backbone, facilitating feature fusion and enhancement. Neck enhances the model’s multi-scale detection capability and accuracy. The detection Head derives the final detection result, including the bounding box, category, and confidence.

**Figure 2:**
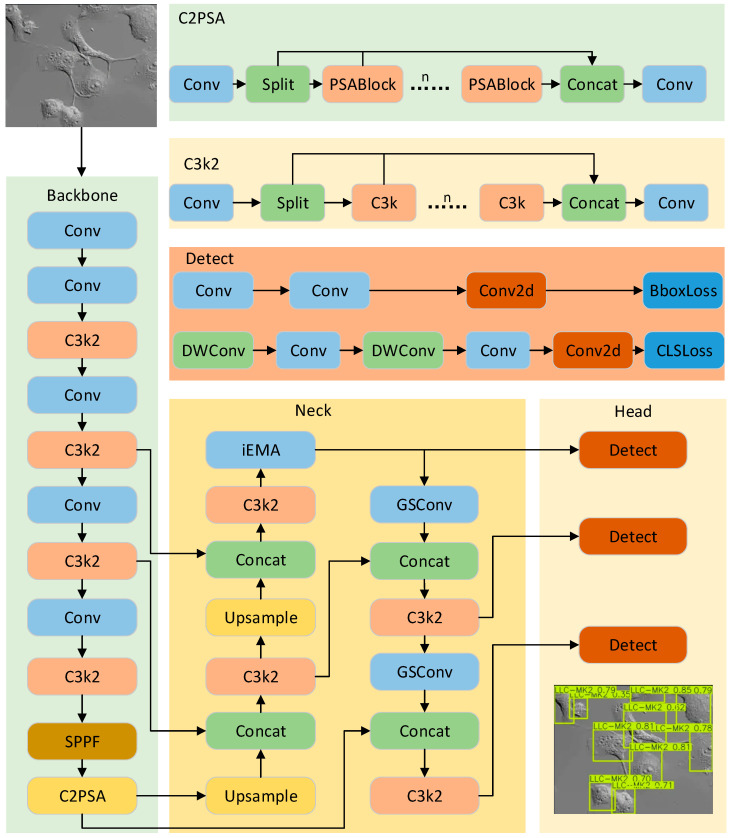
The structure of IEGS-YOLO.

The IEGS-YOLO architecture shown in Figure 2 is similar to YOLO11 architecture. It integrates two novel modules: IEMA and GSConv. The IEMA module uses the inverted residual structure of iRMB in combination with the EMA module to improve model accuracy and robustness, increase model stability and precision in small object detection. It is inserted into the P3 branch of the Neck of Figure 2. The P3 branch is responsible for detecting small objects, which often exhibit severe deformation and low resolution. At this stage, the input feature map has a spatial size of 80 × 80 with 256 channels, assuming an input image size of 640 × 640.

GSConv is used in the feature fusion branches corresponding to P4 and P5 feature maps. It replaces traditional convolution layers in the neck of Figure 2. Specifically, GSConv reduces the computational cost while maintaining sufficient accuracy.

### 2.2. IEMA

**Figure 3:**
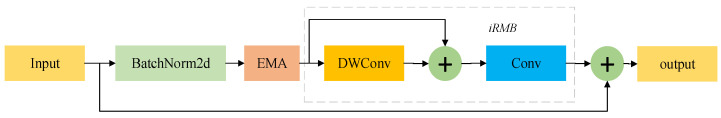
The flow chart of IEMA.

In this section, we propose the IEMA module, a novel architecture developed by integrating the inverted residual connection concept from iRMB into the EMA module, and its flowchart is shown in Figure 3.

Initially, the input data is processed by the BatchNorm2d layer to ensure its normalization. Then, the data is directed to the EMA module, which serves to strengthen the global dependence on the features and enhance the ability to capture local details through multi-scale feature integration and cross-space learning mechanisms. Subsequently, spatial features are extracted using DWConv, while residual concatenation is employed to sum the EMA processed features with the deep convolution processed features. The summed features then flow into the second convolutional layer. Finally, the output of the second convolutional layer is fused with the original input by residual concatenation to output the final processed features. The EMA and iRMB are key modules of the IEMA module. These two submodules are described in Section 2.2.1 and Section 2.2.2 separately.

#### 2.2.1. EMA

**Figure 4:**
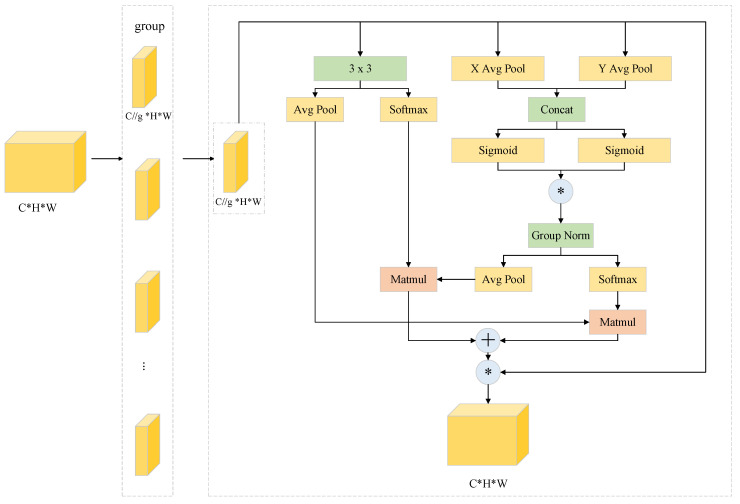
The structure of EMA.

EMA is an efficient multi-scale attention module without dimensionality reduction, which utilizes a channel grouping strategy to reconstruct part of the channel dimensions into batch dimensions, avoiding the information loss of the traditional dimensionality reduction, and its structure is shown in Figure 4. The “g” denotes the number of groups, “X Avg Pool” denotes one-dimensional horizontal average pooling, and “Y Avg Pool” denotes one-dimensional vertical average pooling.

The EMA module is divided into three distinct branches, two of which use a 1 × 1 convolutional branch, and the third uses a 3 × 3 convolutional branch. First, the input feature maps are divided into g groups, and each group of features is pooled by horizontal and vertical global average pooling in a 1 × 1 branch, respectively, to generate compressed features and concatenate them along the channel dimension. Then, the features are fused by a 1 × 1 convolutional branch, and the sigmoid function is applied to generate new channel attention maps, and two-channel attention maps within each group are aggregated by simple multiplication. In the 3 × 3 branch, 3 × 3 convolution is employed to capture local spatial context information to expand the feature space. In addition, cross-dimensional interactions facilitate the aggregation of the outputs of the two parallel branches, capturing pairwise pixel relationships at the pixel level. The final output of the EMA is the same size as the input and can be efficiently incorporated into modern architectures.

#### 2.2.2. iRMB

**Figure 5:**
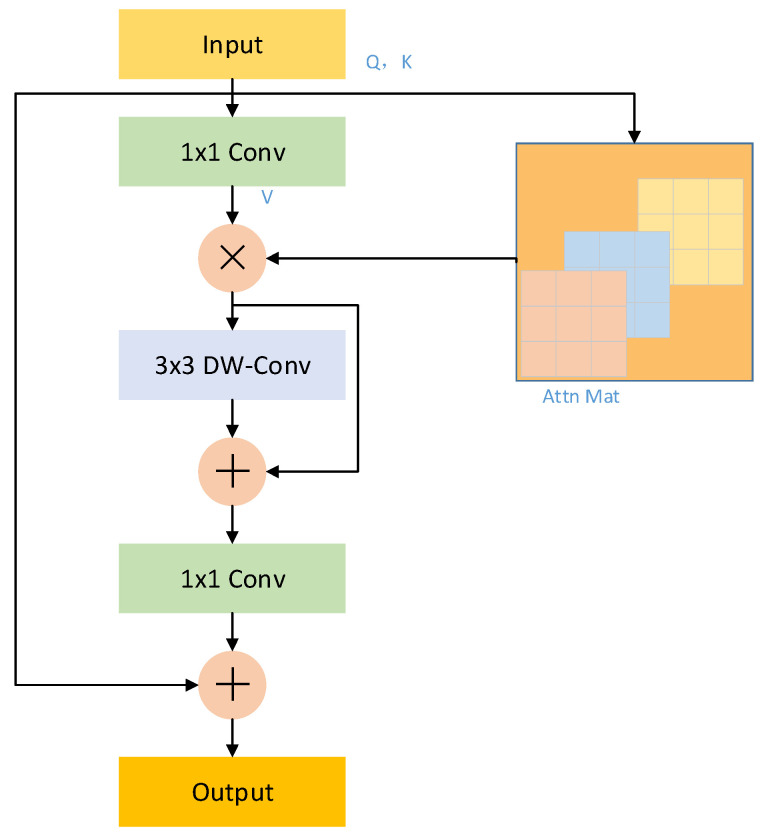
The structure of iRMB.

The iRMB is used to integrate a lightweight convolutional neural network architecture with an attention-based model. By rethinking the inverted residual block (IRB) in MobileNetv2 and two effective modules of transformer—multi-head self-attention (MHSA) and feedforward neural network (FFN), the meta mobile block (MMB) is proposed. The Inverted Residual Mobile Block (iRMB) is instantiated based on the MMB, and its structure is shown in Figure 5.

Initially, the low-dimensional features are expanded into a higher-dimensional space using a 1 × 1 convolution. Then, depthwise separable convolution extracts spatial features, and finally, another 1 × 1 convolution compresses the features back to a low-dimensional representation. Additionally, the self-attention mechanism is employed to model global dependencies.

The MMB is abstracted as a one-residual structure with configurable parameters (expansion ratio and efficient operator) to instantiate different modules. In MMB, the efficient operator F(⋅) is modeled as a cascade operation of MHSA and convolution (Conv), formulated as in Equation (1):

(1) F(⋅)=Conv(MHSA(⋅))

However, this implementation may incur a substantial computational overhead. To address this challenge, the expanded window multi-head self-attention (EW-MHSA) and deep convolution with jump connections (DW-Conv) are employed in iRMB to minimize the computational cost of the model. Accordingly, the efficient operator F(⋅) in iRMB is formulated as shown in Equation (2):

(2) F(⋅)=(DW−Conv,Skip)(EW−MHSA(⋅))

Existing Transformer-based models generally implement module combinations through a layered design. In contrast, iRMB requires only two switches to enable or disable MHSA/DW−Conv.

### 2.3. GSConv

In this study, group shuffle convolution (GSConv), a lightweight convolution, is introduced as a replacement for the traditional convolution in neck networks. GSConv overcomes the disadvantage of channel information separation during deep convolution computation and effectively combines the advantages of standard convolution (SC) and depth-wise separable convolution (DSC) to reduce the computational cost while maintaining sufficient accuracy.

Specifically, GSConv performs a uniform exchange of local feature information among different channels by introducing the shuffle operation on the high-quality features generated by SC and DSC. The computational complexity formulas for SC and GSConv are Equation (3) and Equation (4), respectively.

(3) $$T_{SC}=W\times H\times K_{1}\times K_{2}\times C_{1}\times C_{2}$$

(4) $$T_{GSConv}=W\times H\times K_{1}\times K_{2}\times \frac{C_{2}}{2}\times (C_{1}+1)$$

where W and H represent the width and height of the output feature map, $K_{1}\times K_{2}$ denotes the kernel size of convolution, $C_{1}$ is the number of channels of the input feature map, and $C_{2}$ is the number of channels of the output feature map. From Equations (3) and (4), we can observe that the computational cost of GSConv was about half that of SC.

**Figure 6:**
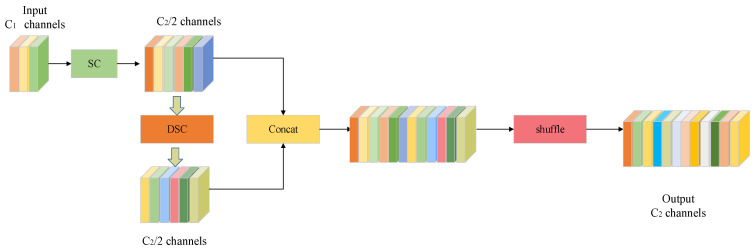
The structure of GSConv.

As shown in Figure 6, first, GSConv performs a downsampling operation on the input feature map using SC and then applies DSC to process the features independently on a channel-by-channel basis. Afterward, the two feature maps generated by the SC and the DSC are concatenated, and the information from the SC is integrated into the output of the DSC through the shuffle, which improves the cross-channel feature interaction capability and effectively reduces the computational cost at the same time.

### 2.4. BoT-SORT

**Figure 7:**
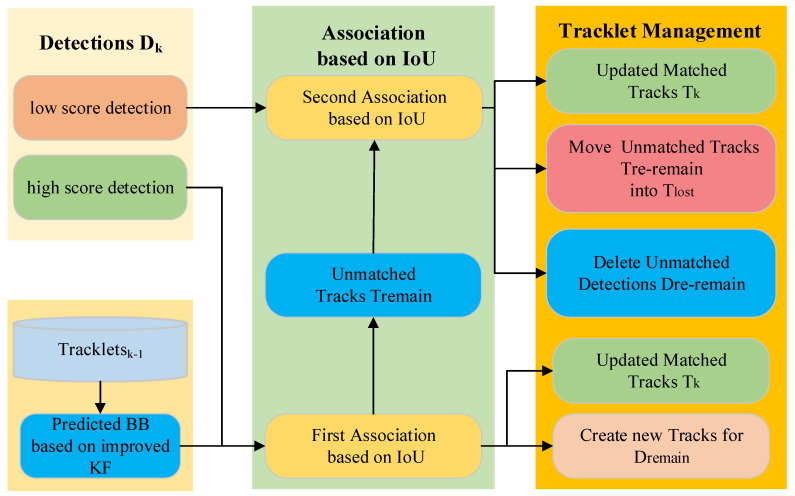
The matching process of BoT-SORT.

The BoT-SORT algorithm retains the low-scoring detection frames with two rounds of matching while ensuring the tracking of the high-scoring detection frames. BoT-SORT mainly consists of the improved Kalman filter (KF), data association, and tracklet management, and its structure is shown in Figure 7.

The improved Kalman filter is first used to predict the cell motion trajectory, which mainly predicts the state of the current frame based on the state of the previous frame of the tracking trajectory, as shown in Equations (5) and (6):

(5) $${\hat{x}}_{k|k-1}=F_{k}{\hat{x}}_{k-1|k-1}$$

(6) $$P_{k|k-1}=F_{k}P_{k-1|k-1}F_{k}^{T}+Q_{k}$$

where ${\hat{x}}_{k|k-1}$ denotes the prior estimate of state at frame *k*, ${\hat{x}}_{k-1|k-1}$ represents the posterior state estimation at frame *k* − 1, $F_{k}$ is the state transition matrix, $P_{k|k-1}$ denotes the covariance matrix of the prior estimate of state at frame *k*, $P_{k-1|k-1}$ represents the covariance matrix of the posterior state estimation at frame *k* − 1, and $Q_{k}$ is the noise covariance matrix.

The data association part utilizes a hierarchical matching strategy to realize the precise association between detection boxes and tracklets. It is categorized into high and low score detection boxes based on the detection score and also includes a collection of trajectories from the previous frame. They will be used for two rounds of data association. The first association is performed between the high-score detection boxes $D_{high}$ and all trajectories. The similarity is calculated by the IoU distances between them. The second association is performed between the unmatched trajectories $T_{remain}$ from the first association and low-score detection boxes $D_{low}$ based on IoU. Through two rounds of association, matched trajectories, unmatched detection boxes, and unmatched trajectories are obtained. For the matched trajectories $T_{k}$, they are updated using the Kalman filter, as shown in Equations (7)–(9).

(7) $$K_{t}={\hat{P}}_{t}H^{T}{\left(H{\hat{P}}_{t}H^{T}+R\right)}^{-1}$$

(8) $$x_{t}={\hat{x}}_{t}+K_{t}(Z_{t}-H{\hat{x}}_{t})$$

(9) $$P_{t}={\hat{P}}_{t}-K_{t}H{\hat{P}}_{t}$$

where $K_{t}$ denotes Kalman gain, H represents the observation matrix, R is the measurement noise covariance matrix, $x_{t}$ is the posterior state estimation at frame *t*, $Z_{t}$ indicates a measured value, and $P_{t}$ is the covariance matrix for the posterior state estimation at frame *t*.

Based on the matching results, the tracklet management module applies a differentiated processing strategy. For the first association’s mismatched detection boxes $D_{remain}$, create new tracks and assign them new IDs. For the second association’s mismatched detection boxes $D_{re-remain}$, delete them. As for the second association’s mismatched tracks $T_{re-remain}$, mark them as lost tracks $T_{lost}$ and delete them if they exceed the preset number of frames.

## 3. Experiments

This section will present the experimental details, the experimental results of IEGS-YOLO + BoT-SORT (IEGS-BoT), and the comparison with other models.

### 3.1. Dataset

This study utilized the CTMC dataset, collected from Nikon Microscopy’s website. The dataset consists of 47 videos with 80,389 frames. These videos have a resolution of 320 × 400 and a frame rate of 30 frames per second. The dataset contains 14 different cell lines, which are described in Table 1. The videos were divided into a training set (40,187 images) and a testing set (40,202 images) for model training and testing according to a ratio of 1:1.

In addition, to assess the robustness of our approach, we also employed an additional publicly available cell detection dataset [32] for supplementary evaluation. This dataset contains 500 images with a resolution of 2048 × 1536 pixels and includes three categories: red blood cells, white blood cells, and crystals.

The data in the study were obtained from publicly available datasets. The results were generated using PyTorch 1.12 and official evaluation scripts from the benchmark datasets.

### 3.2. Evaluation Metrics and Implementation Details

In this study, we utilize standard evaluation metrics in object detection: precision (P), recall (R), F-Measure (F1), mean average precision (mAP), parameters (Parms), model size (size), and Giga Floating Point Operations (GFLOPs) for evaluating detector performance. Precision (P), Recall (R), F1, and mAP indicate the detector’s overall performance. Params focus on quantifying the model’s complexity, with all reported values representing the absolute number of trainable parameters. Model size focuses on the detector’s efficiency, and GFLOPs reflect the computational complexity of the model.

The multi-object tracking part uses higher order tracking accuracy (HOTA), MOT accuracy (MOTA), identification F1 (IDF1), and ID switch (IDSW) to evaluate the tracking method. Among them, MOTA is used to evaluate the overall performance of the multi-object tracking algorithm, HOTA is a comprehensive metric that measures both detection and association accuracy, IDF1 reflects the performance of the tracking algorithm in recognizing the identity of the object, while IDSW is the number of ID switching times in the tracking process, which indicates the stability of ID matching.

For the detection experiment, the parameters are set as follows: epoch is 60, the batch size is 16, and the learning rate is 0.01. For the tracking experiment, the parameters are set as follows: the confidence thresholds for the first and second associations are 0.5 and 0.1, the confidence threshold for initializing new trajectories is 0.6, and the threshold for association matching is 0.8.

### 3.3. Ablation Experiments with IEGS-YOLO

Before conducting the ablation study, we performed a series of hyperparameter tuning experiments for the YOLO11n detector to ensure a strong baseline. As shown in Table 2, the model performs best when using AdamW with batch size 16 and learning rate 0.01.

To validate the effectiveness of each module under the selected hyperparameters, we conduct the following ablation study. The ablation study for YOLO11n with and without IEMA and GSConv is presented in Table 3. From Table 3, we can observe that the baseline obtained a precision of 76.9%, recall of 69.1%, F1 score of 72.8%, map50 of 72.1%, and map50–95 of 32.0%. The model has 2,584,882 parameters, with a model size of 5.5 MB and a computational cost of 6.3 GFLOPs. The incorporation of the IEMA module improves recall by 0.7%, F1 by 0.2%, mAP@0.50 by 1.0%, and mAP@0.50–0.95 by 0.7%, while the parameters and computational cost show a slight increase. The introduction of the GSConv module improves precision, recall, F1, and map50 by 0.1%, 0.5%, 0.3%, and 0.5%, respectively, with the lowest number of parameters, model size, and computational complexity.

Overall, the GSConv module maintains excellent performance while reducing model complexity, while the IEMA mechanism is more appropriate for scenarios where high recall and detection robustness are required. When both GSConv and IEMA are incorporated, the mAP50 increases to 73.2% and mAP50–95 reaches 32.6%, nearly matching the accuracy achieved by introducing the IEMA module alone. Meanwhile, the parameters, model size, and computational cost remain close to those of using GSConv individually. This fused architecture consequently emerges as an optimal solution that balances both accuracy and efficiency.

**Figure 8:**
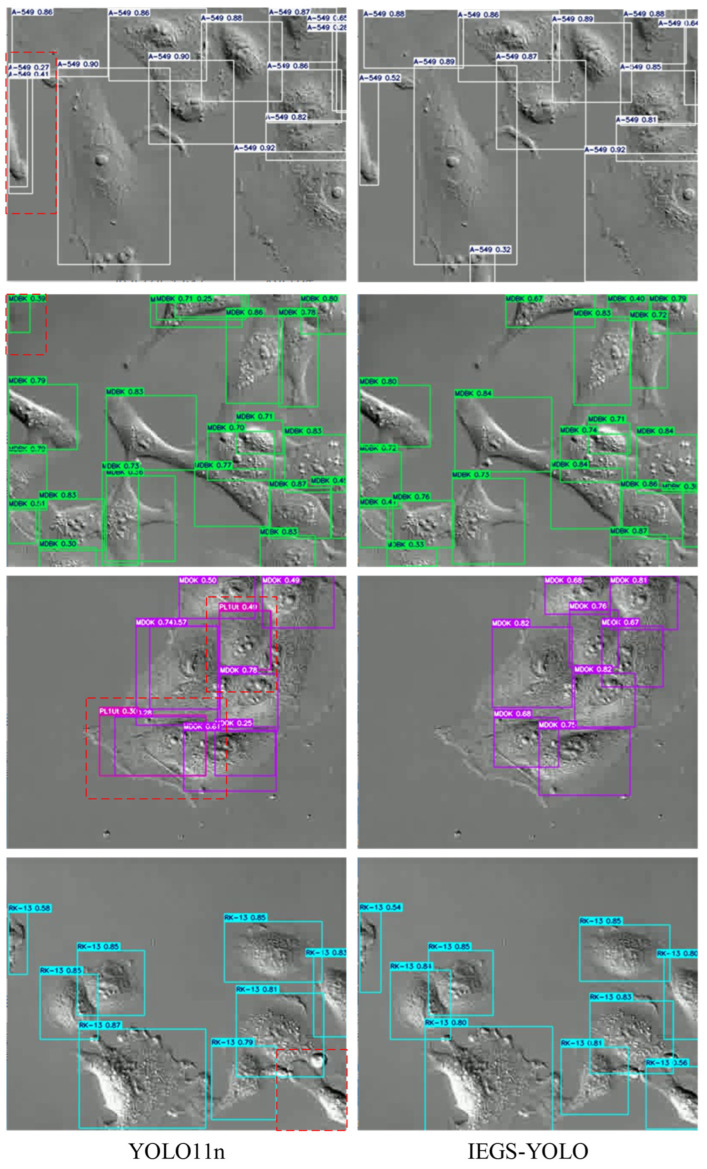
The detection results of YOLO11n and IEGS-YOLO.

Figure 8 shows the comparison of the detection results of YOLO11n, and the IEGS-YOLO method proposed in this study on four different categories of cells. Where the left column is the detection result of YOLO11n, the right column is the detection result of the IEGS-YOLO method, and the red dashed box is the area we need to focus on. As illustrated in Figure 8, the enhanced detector proposed in this study effectively mitigates the issues of missed detection and false detection of cells under complex backgrounds. In the first row, the YOLO11n model incorrectly identifies a single target as two distinct targets, while the enhanced detector accurately detects a single target. In the second row, YOLO11n misjudges the background as one target, while the improved detector avoids this error. In the third row, YOLO11n incorrectly identifies the cell category MDOK as PL1Ut, while the proposed detector accurately identifies the category. In the fourth row, YOLO11n misses a target during detection, while IEGS-YOLO correctly detects the target.

To verify the generalization of our method, we selected another publicly available cell image detection dataset to conduct a supplementary evaluation of detection performance, comparing YOLO11n with our proposed IEGS-YOLO model. As shown in Table 4, IEGS-YOLO achieved a significant improvement in precision, increasing from 82.0% to 88.1%, and an increase in F1 score and Map50. Additionally, IEGS-YOLO further reduces the number of model parameters and memory usage without increasing computational costs, achieving a better balance between accuracy and efficiency. These results validate that our method maintains detection accuracy while achieving higher efficiency.

#### 3.3.1. Comparison of the Performance of Different Attention Mechanisms

Table 5 demonstrates the comparison results of the YOLO11n model after incorporating different attention mechanisms. After adding the IEMA module, recall, F1 score, map50, and map50–95 reached 69.8%, 73.0%, 73.1%, and 32.7%, respectively, which represent significant improvements over the baseline. In contrast, the MLCA attention mechanism [33] showed a decrease in most metrics, and the MSDA attention mechanism [34] did not improve as much as IEMA, although it showed a small increase in some metrics. Therefore, we chose the IEMA module.

#### 3.3.2. Comparison of the Performance of Different Convolutional Modules

We also comprehensively investigated the effect of different convolutional modules on model performance, as shown in Table 6. After incorporating the GSConv module, precision, recall, F1 score, and map50 increased to 77.0%, 69.6%, 73.1%, and 72.6%, respectively, while the number of parameters, model size, and computational cost were all reduced, which reflects its dual advantages in enhancing performance and reducing model complexity. In contrast, the integration of the MAB module [35] resulted in worse detection performance. Meanwhile, while the PConv module [36] achieved reductions in parameter count, model size, and computational complexity, its detection performance remained inferior to that of the YOLO11n.

Overall, the MAB module remains inferior to the YOLO11n both in terms of detection performance and resource consumption, and the PConv is not as good as the YOLO11n, although it has reduced resource consumption. The GSConv module improves the detection performance and reduces the model complexity at the same time, which is the best choice for the balance between efficiency and accuracy.

### 3.4. Comparison of the Performance of Different Detection Algorithms

Table 7 presents the detection results based on different detectors. The suffixes in YOLO series refer to the different model sizes: “n” stands for nano, “s” for small, “m” for medium, and “l” for large. These variants share the same core architecture and backbone design but differ in network width and depth. This naming convention aligns with the usage introduced in previous YOLO versions, except for YOLOv9t [37], where “t” represents “tiny.”

We can observe that the detectors differ in accuracy and complexity. IEGS-YOLO achieves the best performance in precision, recall, F1 score, and mAP50, while it is slightly inferior to YOLO11s and YOLO11m in mAP50–95. We acknowledge that both YOLO11m and YOLO11s achieve slightly higher mAP@0.50–0.95 than IEGS-YOLO, but it is important to note that both of these models exhibit larger model sizes, higher parameter counts, and greater computational costs compared to IEGS-YOLO. Our work aims to balance detection accuracy and efficiency, especially under resource-constrained conditions such as real-time clinical applications. Based on all metrics, we find that IEGS-YOLO performs better than YOLO11m and YOLO11s overall, particularly when considering factors such as precision, recall, and F1 score, while maintaining a significantly lower computational cost.

Compared to other lightweight versions of YOLO, YOLO11n outperforms YOLOv5n, YOLOv8n, YOLOv9t, and YOLOv10n [38] in terms of precision, recall, F1 scores, map50, and map50–95. Overall, IEGS-YOLO achieves the best performance while maintaining a low model complexity and computational cost.

### 3.5. Comparison of the Performance of Different Tracking Algorithms

Table 8 presents a comparison of tracking results in test videos for our method, TransTrack [39], DeepSORT [40], ImprAsso [41], OC-SORT [42], Deep OC-SORT [43], ByteTrack [44], and BoT-SORT. It is observed that our method outperforms other tracking algorithms in most metrics. From the perspectives of MOTA, HOTA, and IDF1, IEGS-BoT stands out with scores of 53.97%, 51.30% and 67.52%, respectively. Compared to TransTrack, DeepSORT, ImprAsso, OC-SORT, Deep OC-SORT, ByteTrack, and BoT-SORT, our method achieves improvements in MOTA by 2.41%, 9.16%, 7.26%, 12.68%, 7.34%, 1.53% and 1.19%; in HOTA by 6.88%, 8.7%, 12.71%, 10.69%, 8.04%, 0.27% and 0.23%; and in IDF1 by 11.34%, 13.35%, 22.66%, 12.55%, 10.36%, 1.15% and 1.29%, respectively. Furthermore, our method shows the best performance in IDSW with only 894, which is significantly lower than 5985, 2938, 13,107, 1427, 2758, 1217 and 1170 obtained by TransTrack, DeepSORT, ImprAsso, OC-SORT, Deep OC-SORT, ByteTrack, and BoT-SORT, respectively. Overall, our method achieves the best results in MOTA, HOTA, IDF1, and IDSW, particularly in IDSW, which demonstrates the superior identity consistency of our approach, leading to more stable and reliable tracking performance.

**Figure 9:**
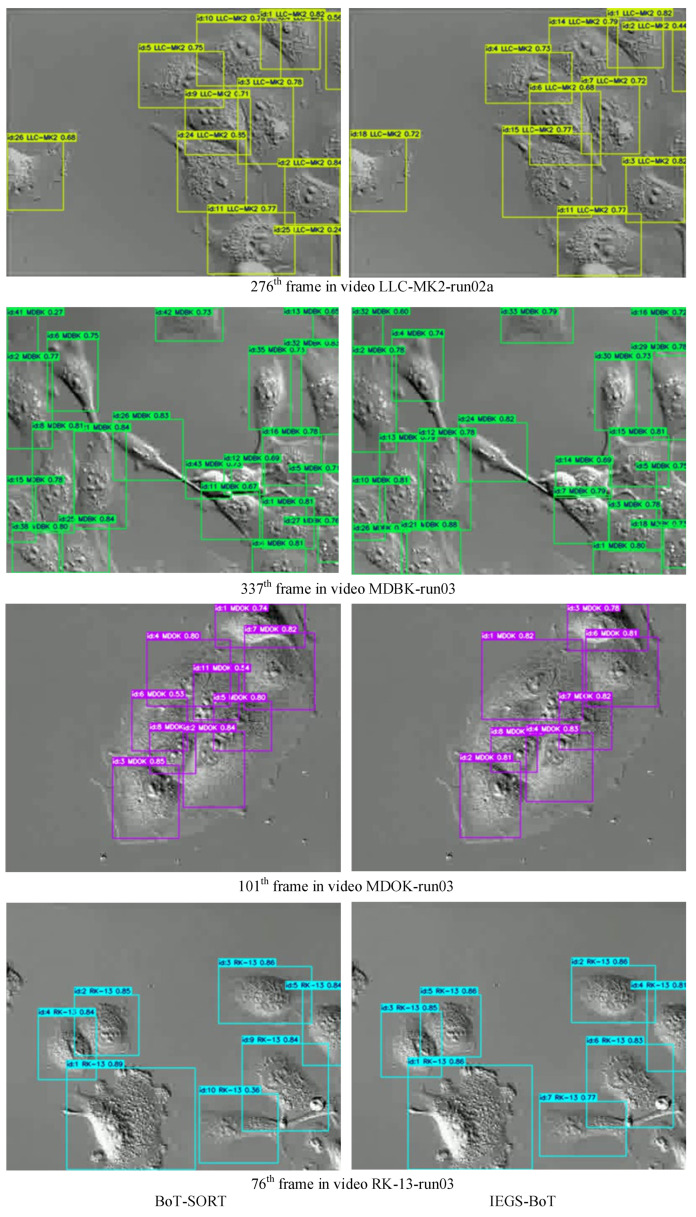
The tracking results of BoT-SORT and IEGS-BoT.

Figure 9 illustrates the comparison of tracking performance between BoT-SORT and IEGS-BoT in four different video sequences. The left column presents the tracking visualization results of BoT-SORT, while the right column shows the corresponding visualizations of the IEGS-BoT. Experimental results indicate that our method has significant advantages in maintaining identity (ID) consistency. The ground truth ID counts for the four test video sequences are 18, 25, 8 and 7, respectively. In contrast, BoT-SORT yielded a maximum ID count of 26, 43, 11 and 10, while IEGS-BoT generated only 18, 33, 8 and 7 IDs, which align more closely with the real ID numbers. These quantitative results demonstrate that IEGS-BoT effectively suppresses the occurrence of cross-frame ID switching, significantly enhancing the stability of the tracking process.

**Figure 10:**
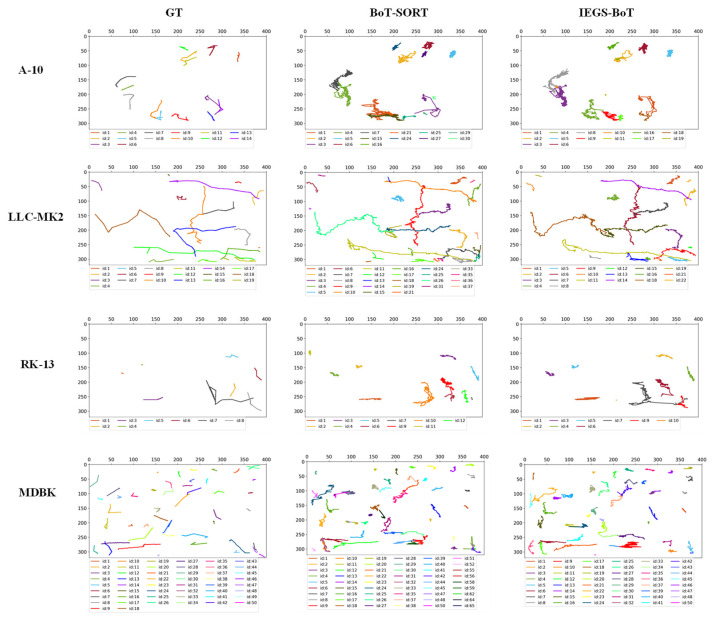
Comparison of Cell Trajectories using IEGS-BoT and BoT-SORT.

To further compare the overall tracking performance among different trackers, Figure 10 displays the real trajectories of all cells in selected video sequences, along with the tracking trajectories of BoT-SORT and our proposed IEGS-BoT method.

The ground truth (GT) of actual cell trajectories is obtained through frame-by-frame manual annotations in the video sequences. Each annotation includes the frame, ID, and the bounding box coordinates (left, top, width, height). The cell trajectories are defined as sequences of bounding boxes sharing the same ID across consecutive frames. These annotations are derived from a public dataset and follow the standardized MOTChallenge format, ensuring high accuracy and consistency for reliable performance evaluation.

Compared with BoT-SORT, our method demonstrates improvements in trajectory overlap with the ground truth, with particularly significant improvements in identity preservation, reflected in fewer ID switches and more continuous and stable trajectories. These results suggest that the combination of IEGS-YOLO and BoT-SORT offers stronger identity preservation in complex cellular motion scenarios. These results further validate the effectiveness of this method for cell tracking tasks.

## 4. Discussion

In this section, we discuss the limitations of our approach, highlighting the key challenges and providing insights into areas that can be improved in future work.

Although our method demonstrates strong performance in cell tracking, it faces limitations when dealing with certain cell types that are difficult to detect accurately, such as APM cells. In particular, the APM sequence presents a challenging case due to significant shape deformation across frames, despite containing relatively few cells. Compared with other cell types, APM cells are more likely to be confused with the background. This leads to missed detections, false detections, and identity switches.

**Figure 11:**
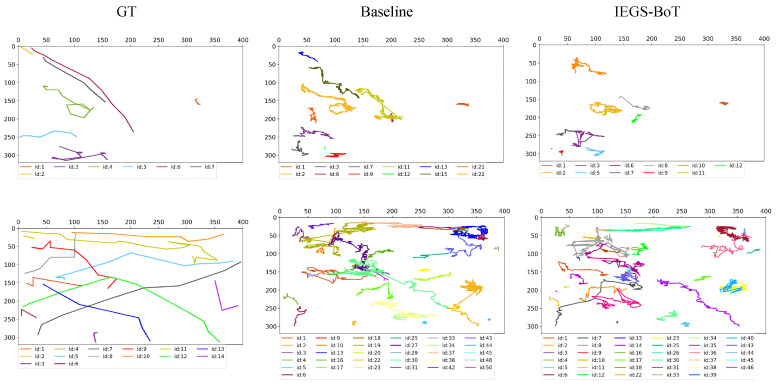
Comparison of Cell Trajectories on APM Sequence.

We compare the GT of actual cell trajectories with the trajectories generated by the baseline method and our improved method on the APM sequence. As shown in Figure 11, our method demonstrates some improvement over the baseline in reducing ID switches. However, a clear gap remains compared to the ground truth, indicating that our method still has limitations when dealing with such complex scenarios.

We believe this example effectively illustrates the practical challenges our model faces and provides meaningful guidance for future improvements. Addressing such difficult cases will be an important direction in our future research.

## 5. Conclusions

In this study, we proposed an improved MOT method, IEGS-BoT (IEGS-YOLO + BoT-SORT). The approach aimed to solve the problems of missed detection, false detection, and identity switching in the microscopic scenario. These issues are caused by challenging factors such as ambiguous boundaries, background interference, high cell morphology similarity, and irregular division.

To achieve the best object detection performance, we conducted comparative analyses of detectors like YOLOv5, YOLOv8, YOLOv9, YOLOv10, and YOLO11, ultimately selecting YOLO11n as the baseline, based on which we proposed IEGS-YOLO. Firstly, we introduced an innovative iEMA module in the small object detection layer to enhance the model’s accuracy in detecting small targets. Secondly, we replaced the traditional convolution in the neck network with GSConv, a module that reduced computational complexity while maintaining accuracy. The proposed detector obtained a precision of 77.0%, recall of 69.6%, F1 score of 73.1%, mAP@0.50 of 73.2%, mAP@0.50–0.95 of 32.6% on the CTMC dataset, and the model size was only 5.3 MB.

In cell tracking, we compared tracking algorithms including TransTrack, DeepSORT, ImprAsso, OC-SORT, Deep OC-SORT, ByteTrack, and BoT-SORT. The results demonstrated that BoT-SORT achieved the best performance. When integrated with the improved detector, it achieved 51.30% of HOTA, 53.97% of MOTA, and 67.52% of IDF1, respectively. In addition, the IDSW was only 894, ensuring more reliable cell tracking.

## Figures and Tables

**Table 1:** Description of each cell line.

Label	Description
3T3	Albino Swiss Mouse Embryo—fibroblasts
A-10	Rat Thoracic Aortic Mesenchyme-myoblasts
A-549	Male Human Lung Cancer—epithelial
APM	African Otter Skin—fibroblasts
BPAE	Bovine Pulmonary Artery—epithelial
CV-1	Normal African Green Monkey Kidney—fibroblasts
CRE-BAG2	Albino Swiss Mouse Embryo Moroni Murine Leukemia Virus Transfected Cells—fibroblasts
LLC-MK2	Rhesus Monkey Kidney—epithelial
MDBK	Madin-Darby Bovine Kidney—epithelial
MDOK	Madin-Darby Ovine Kidney—epithelial
OK	Opossum Kidney Cortex Proximal Tubule—epithelial
PL1Ut	Raccoon Uterus—fibroblasts
RK-13	Normal Rabbit Kidney—epithelial
U2O-S	Human Bone Osteosarcoma—epithelial

**Table 2:** Comparison of results of different hyperparameters (Best metric values are shown in bold).

Optimizer	Batch Size	LR	P (%)	R (%)	F1 (%)	Map50 (%)	Map 50–95 (%)
AdamW	16	0.01	**76.9**	**69.1**	**72.8**	**72.1**	32.0
AdamW	16	0.001	75.8	67.6	71.5	70.4	31.4
AdamW	32	0.01	76.5	68.4	72.2	71.9	**32.2**
AdamW	32	0.001	74.0	65.7	69.6	68.7	30.9
SGD	16	0.01	75.2	66.2	70.4	69.9	31.5
SGD	16	0.001	73.1	63.4	67.9	67.1	28.5
SGD	32	0.01	76.2	64.8	70.0	69.3	31.2
SGD	32	0.001	72.0	61.1	66.1	65.9	28.2

**Table 3:** Ablation experiments with different modules (Best metric values are shown in bold).

	P (%)	R (%)	F1 (%)	Map50 (%)	Map 50–95 (%)	Params	Size (MB)	GFLOPs (G)
YOLO11n	76.9	69.1	72.8	72.1	32.0	2,584,882	5.5	6.3
+IEMA	76.5	**69.8**	73.0	73.1	**32.7**	2,589,858	5.5	6.4
+GSConv	**77.0**	69.6	**73.1**	72.6	32.0	**2,495,314**	**5.3**	**6.2**
IEGS-YOLO (Present)	**77.0**	69.6	**73.1**	**73.2**	32.6	2,500,290	**5.3**	6.3

**Table 4:** Comparison of the performance of YOLO11n and IEGS-YOLO on Cell Image Detection (Best metric values are shown in bold).

	P (%)	R (%)	F1 (%)	Map50 (%)	Map 50–95 (%)	Parms	Size (MB)	GFLOPs (G)
YOLO11n	82.0	**68.6**	74.7	79.4	**46.5**	2,582,737	5.5	6.3
IEGS-YOLO (Present)	**88.1**	65.7	**75.3**	**79.8**	46.4	**2,498,145**	**5.3**	6.3

**Table 5:** Comparison of different attention mechanisms in YOLO11n (Best metric values are shown in bold).

	P (%)	R (%)	F1 (%)	Map50 (%)	Map 50–95 (%)	Params	Size (MB)	GFLOPs (G)
YOLO11n	**76.9**	69.1	72.8	72.1	32.0	**2,584,882**	5.5	**6.3**
+IEMA	76.5	**69.8**	**73.0**	**73.1**	**32.7**	2,589,858	5.5	6.4
+MLCA	76.4	68.3	72.1	72.0	31.9	2,584,888	5.5	**6.3**
+MSDA	76.2	69.4	72.6	72.4	32.5	2,601,522	5.5	6.5

**Table 6:** Comparison of different convolutional modules in YOLO11n (Best metric values are shown in bold).

	P (%)	R (%)	F1 (%)	Map50 (%)	Map 50–95 (%)	Params	Size (MB)	GFLOPs (G)
YOLO11n	76.9	69.1	72.8	72.1	32.0	2,584,882	5.5	6.3
+GSConv	**77.0**	**69.6**	**73.1**	**72.6**	32.0	**2,495,314**	**5.3**	**6.2**
+MAB	76.2	67.4	71.5	70.9	31.9	2,587,922	5.5	6.3
+PConv	75.7	68.7	72.0	71.9	**32.3**	2,555,346	5.4	**6.2**

**Table 7:** Comparison of detection performance of different models (Best metric values are shown in bold).

	P (%)	R (%)	F1 (%)	Map50 (%)	Map 50–95 (%)	Params	Size (MB)	GFLOPs (G)
YOLOv5n	74.1	65.6	69.6	69.3	31.0	2,184,394	4.6	**5.8**
YOLOv8n	75.0	66.0	70.2	70.0	31.8	2,687,098	5.6	6.8
YOLOv9t	75.4	68.7	71.9	71.6	32.5	**1,732,554**	**4.1**	6.5
YOLOv10n	76.1	67.1	71.3	70.2	30.9	2,699,876	5.7	8.3
YOLO11n	76.9	69.1	72.8	72.1	32.0	2,584,882	5.5	6.3
YOLO11s	75.4	65.6	70.2	70.6	**33.4**	9,418,218	19.2	21.3
YOLO11m	75.7	66.0	70.5	70.9	33.0	20,040,826	40.5	67.7
YOLO11l	73.0	60.8	66.3	66.2	31.6	25,290,106	51.2	86.6
IEGS-YOLO (Present)	**77.0**	**69.6**	**73.1**	**73.2**	32.6	2,500,290	5.3	6.3

**Table 8:** Comparison of tracking results of different tracking algorithms (Best metric values are shown in bold).

	MOTA (%)	HOTA (%)	IDF1 (%)	IDSW
TransTrack	51.56	44.42	56.18	5985
DeepSORT	44.81	42.60	54.17	2938
ImprAsso	46.71	38.59	44.86	13,107
OC-SORT	41.29	40.61	54.97	1427
Deep OC-SORT	46.63	43.26	57.16	2758
Bytetrack	52.44	51.03	66.37	1217
Bot-Sort	52.78	51.07	66.23	1170
IEGS-BoT (Present)	**53.97**	**51.30**	**67.52**	**894**

## Data Availability

The raw data supporting the conclusions of this article will be made available by the authors on request.
